# A Review on the Radioprotective Activity of organogermanium and Organosilicon Compounds

**DOI:** 10.1155/MBD.1999.49

**Published:** 1999

**Authors:** Ghassoub Rima, Jacques Satgé, Rodolphe Dagiral, Claude Lion, Henri Sentenac-Roumanou, Marc Fatôme, Vincent Roman, Jean-Denis Laval

**Affiliations:** 1 Laboratoire d'Hétérochimie Fondamentale et Appliquée UPRES-A 5069 du CNRS Université Paul Sabatier 118, route de Narbonne Toulouse Cedex 4 F-31062 France; 2 Institut de Topologie et de Dynamique des Systèmes de I'Universit de Paris VII Associé au CNRS 1, rue Guy de la Brosse Paris F-75005 France; 3 Direction des Recherches Etudes et Techniques 26, boulevard Victor Armées F-00460 France; 4 Unité de Radioprotection Centre de Recherche du Service de Santé des Armées 24, avenue des Maquis du Grésivaudan La Tronche Cedex F-38702 France

## Abstract

The present review describes the work carried out during the last 20 years in the field of the radioprotective activity and toxicity of several classes of organosilicon and organogermanium compounds (i.e. metallathiazolidines, metalladithioacetals, metallatranes and germathianes).


### A REVIEW ON THE RADIOPROTECTIVE ACTIVITY OF ORGANOGERMANIUM AND ORGANOSILICON COMPOUNDS

Ghassoub Rima[1], Jacques Satgé*[1], Rodolphe Dagiral[1], Claude Lion[2], Henri Sentenac-Roumanou[3], Marc Fatôme[4], Vincent Roman[4] and Jean-Denis Laval[4]

[1] Laboratoire d'Hétérochimie Fondamentale et Appliquée, UPRES-A 5069 du CNRS, Université Paul Sabatier, 118, route de Narbonne, F-31062 Toulouse Cedex 4, France

[2] Institut de Topologie et de Dynamique des Systèmes de l'Université de Paris VII, Associé au CNRS, 1, rue Guy de la Brosse, F-75005 Paris, France

[3] Direction des Recherches, Etudes et Techniques, 26, boulevard Victor, F-00460 Armées, France

[4] Unité de Radioprotection, Centre de Recherche du Service de Santé des Armées, 24, avenue des Maquis du Grésivaudan, F-38702 La Tronche Cedex, France

**Abstract**

The present review describes the work carried out during the last 20 years in the field of the radioprotective activity and toxicity of several classes of organosilicon and organogermanium compounds (i.e. metallathiazolidines, metalladithioacetals, metallatranes and germathianes).

### 1. Introduction

During the 20-year period between 1978 and 1998 the Délégation Générale pour l'Armement and Département de Chimie Pharmacologie, France, sponsored a coordinated antiradiation drug development program. The objective of this program was to develop a drug or combination of drugs with organometallated compounds which could be taken by military personnel or other populations to protect them from the effects of ionizing radiations in a nuclear weapons attack, medical treatments or, in general, all radiation exposition.

During the research program approximately 562 compounds were chemically synthesized and tested in mice for their radioprotective properties.

The vast majority of these compounds were metallathiazolidines, metalladithioacetals of *N*-substituted cysteamine, methylcysteamine, *N*-(2-thioethyl)-1,3-diaminopropane, *N*-substituted cysteamine or methylcysteamine by naphthylmethylimidazoline and *N*-substituted naphthylmethylimidazoline.

120 compounds of these derivatives are characterized by a dose reduction factor (DRF) between 1.3 and 1.75.

The radioprotective activity and toxicity of several classes of organometallated derivatives (silathiazolidines, germathiazolidines, siladithioacetals, germadithioacetals, germatranes, silatranes and germylated sulfides) and their synthesis are reported.

All the biological tests have been performed in the Centre de Recherche du Service de Santé des Armées, La Tronche, France.

### 2. Sila- and germathiazolidines

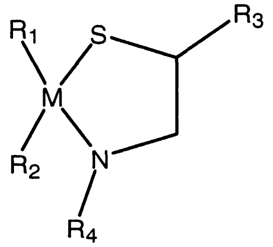

Sila- and germathiazolidines of *N*-substituted cysteamine, methylcysteamine, *N*-(2-thioethyl)-1,3-diaminopropane and *N*-substituted cysteamine or methylcysteamine by naphthylmethylimidazoline were prepared according to two methods of heterocyclisation already described in the literature [1-3].

**Method A**

The action of diorganosilicon and –germanium dichloride [3] (in stoichiometric amounts) on *N*-substituted cysteamine, methylcysteamine, *N*-(2-thioethyl)-1,3-diaminopropane and *N*-substituted cysteamine or methylcysteamine by naphthylmethylimidazoline in refluxing anhydrous tetrahydrofuran in the presence of freshly distilled triethylamine gave by a cyclisation reaction, with elimination of hydrochloric acid from M-Cl and SH and NH [4] groups the corresponding products, Scheme 1.

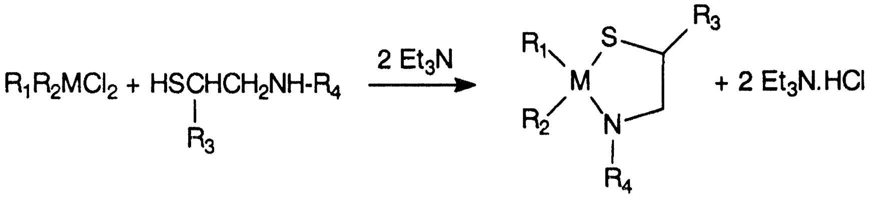

**Scheme 1**

**Method B**

The reaction of *N*-substituted cysteamine, methylcysteamine, *N*-(2-thioethyl)-1,3-diaminopropane and *N*-substituted cysteamine or methylcysteamine by naphthylmethylimidazoline, in stoichiometric amounts, with the bis(diethylamino)dialkylsilanes or –germanes in anhydrous tetrahydrofuran resulted in the cleavage of M-N bonds by the NH and SH groups (a transamination reaction) [1, 3-5], forming the corresponding sila- and germathiazolidines in good yields, Scheme 2.

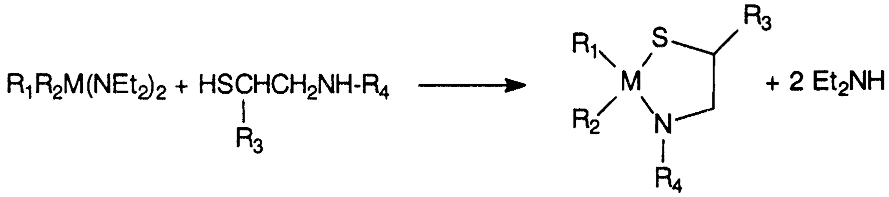

**Scheme 2**

A radioprotective study of metallathiazolidines shows that the silylated and germylated derivatives have a greater biological activity and a lower toxicity than that of the corresponding organic compounds [1] (Table I).

### 3. Sila- and germadithioacetals

$R_1R_2M[S\text{-}CH(R_3)\text{-}CH_2R_4]_2$

Sila- and germadithioacetals of *N*-substituted cysteamine, methylcysteamine, *N*-(2-thioethyl)-1,3-diaminopropane, *N*-substituted cysteamine and methylcysteamine by naphthylmethylimidazoline and *N*-substituted naphthylmethylimidazoline were also obtained by two methods C and D [10].

**Method C**

The action of diorganosilicon and –germanium dichloride [3] on two equivalents of *N*-substituted cysteamine, methylcysteamine, *N*-(2-thioethyl)-1,3-diaminopropane, *N*-substituted cysteamine and methylcysteamine by naphthylmethylimidazoline and *N*-substituted naphthylmethylimidazoline in refluxing anhydrous tetrahydrofuran in the presence of freshly distilled triethylamine gave the acyclic derivatives, Scheme 3.

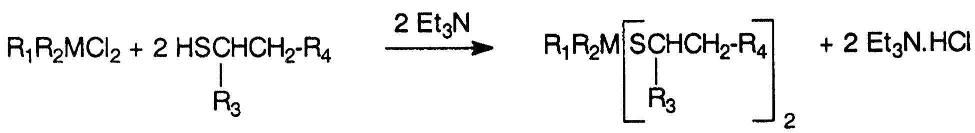

**Scheme 3**

**Table I - Radioprotective activity of some selected metallathiazolidines**

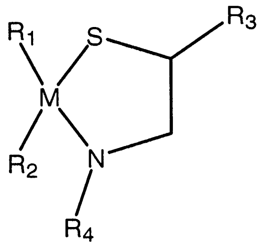

| M = Si | | | | | | | | | |
|---|---|---|---|---|---|---|---|---|---|
| **Cpd** [Ref.] | $R_1$ | $R_2$ | $R_3$ | $R_4$ | $LD_{50}$ ($mg.kg^{-1}$) | Injected dose ($mg.kg^{-1}$) | Irradiation Gy (t, min)[a] | Survival rate % | DRF[b] |
| **1** [1] | $CH_3$ | $C_6H_5$ | H | H | 800 | 400<br>400<br>400<br>100 | 10 (15)<br>10 (120)<br>12 (15)<br>10 (15) | 93<br>90<br>70<br>16 | 1.6 |
| **2** [1] | $CH_3$ | $C_6H_5$ | $CH_3$ | H | 750 | 375<br>375<br>375<br>93.7 | 10 (15)<br>10 (120)<br>12 (15)<br>10 (15) | 95<br>0<br>89<br>75 | 1.5 |
| **3** [1] | $C_2H_5$ | $C_2H_5$ | H | H | 1000 | 500<br>500<br>500 | 9.5 (15)<br>9.5 (120)<br>11.5 (15) | 80<br>90<br>50 | 1.4 |
| **4** [1] | $CH_3$ | *p*-$CH_3C_6H_4$ | H | H | 1500 | 750<br>750<br>750<br>187 | 9.5 (15)<br>9.5 (120)<br>11.5 (15)<br>9.5 (15) | 90<br>70<br>60<br>0 | 1.4 |
| **5** [1] | $CH_3$ | *p*-$CH_3OC_6H_4$ | H | H | 1500 | 750<br>750<br>750<br>187 | 9.5 (15)<br>9.5 (120)<br>11.5 (15)<br>9.5 (15) | 95<br>60<br>50<br>27 | 1.4 |
| **6** [6] | $HCl.H_2N(CH_2)_2S$ | $HCl.H_2N(CH_2)_2S$ | H | (c) | 600 | 300<br>300<br>300<br>75 | 7.5 (15)<br>7.5 (90)<br>9.5 (15)<br>7.5 (15) | 100<br>50<br>90<br>30 | 1.6 |
| **7** [7] | *n*-$C_6H_{13}$ | *n*-$C_6H_{13}$ | H | (d) | 250 | 125<br>125<br>125<br>31.25 | 8 (15)<br>8 (90)<br>10 (15)<br>8 (15) | 100<br>80<br>0<br>0 | 1.3 |
| **8** [7] | *n*-$C_6H_{13}$ | *n*-$C_6H_{13}$ | $CH_3$ | (d) | 220 | 110<br>110<br>110<br>27.5 | 8 (15)<br>8 (90)<br>10 (15)<br>8 (15) | 80<br>90<br>0<br>50 | 1.3 |

a: t = time between administration of compound and irradiation.
b: dose reduction factor = ($LD_{50(30\ days)}$ treated/$LD_{50(30\ days)}$ untreated).

c: $R_4$ =

d: $R_4$ =

| M = Ge | | | | | | | | | |
|---|---|---|---|---|---|---|---|---|---|
| Cpd [Ref.] | $R_1$ | $R_2$ | $R_3$ | $R_4$ | $LD_{50}$ ($mg.kg^{-1}$) | Injected dose ($mg.kg^{-1}$) | Irradiation Gy (t, min)[a] | Survival rate % | DRF[b] |
| 9 | $CH_3$ | $C_6H_5$ | H | H | 600 | 300 | 9.5 (15) | 100 | 1.6 |
| [1] | | | | | | 300 | 11.5 (15) | 70 | |
| | | | | | | 300 | 13.5 (15) | 40 | |
| | | | | | | 75 | 9.5 (15) | 50 | |
| 10 | $CH_3$ | $C_6H_5$ | $CH_3$ | H | 500 | 250 | 9.5 (15) | 80 | 1.75 |
| [1] | | | | | | 250 | 9.5 (120) | 0 | |
| | | | | | | 250 | 11.5 (15) | 70 | |
| | | | | | | 250 | 13.5 (15) | 70 | |
| | | | | | | 250 | 15.5 (15) | 0 | |
| | | | | | | 62.5 | 9.5 (15) | 5 | |
| 11 | $CH_3$ | $p$-$CH_3OC_6H_4$ | H | H | 700 | 350 | 9.5 (15) | 100 | 1.45 |
| [1] | | | | | | 350 | 9.5 (120) | 20 | |
| | | | | | | 350 | 11.5 (15) | 70 | |
| | | | | | | 87.5 | 9.5 (15) | 13 | |
| 12 | $CH_3$ | $p$-$CH_3OC_6H_4$ | $CH_3$ | H | 600 | 300 | 9.5 (15) | 95 | 1.45 |
| [1] | | | | | | 300 | 9.5 (120) | 20 | |
| | | | | | | 300 | 11.5 (15) | 70 | |
| | | | | | | 75 | 9.5 (15) | 20 | |
| 13 | $C_6H_5$ | $C_6H_5$ | H | H | 400 | 200 | 9.5 (15) | 90 | 1.4 |
| [1] | | | | | | 200 | 11.5 (15) | 30 | |
| | | | | | | 100 | 9.5 (15) | 70 | |
| 14 | $n$-$C_4H_9$ | $n$-$C_4H_9$ | H | H | 700 | 350 | 9.5 (15) | 80 | 1.45 |
| [1] | | | | | | 350 | 9.5 (45) | 30 | |
| | | | | | | 350 | 11.5 (15) | 70 | |
| 15 | $n$-$C_4H_9$ | $n$-$C_4H_9$ | $CH_3$ | H | 300 | 150 | 9.5 (15) | 100 | 1.4 |
| [1] | | | | | | 150 | 9.5 (90) | 90 | |
| | | | | | | 150 | 11.5 (15) | 30 | |
| | | | | | | 37.5 | 9.5 (15) | 80 | |
| 16 | $n$-$C_5H_{11}$ | $n$-$C_5H_{11}$ | H | H | 1000 | 500 | 9.5 (15) | 80 | 1.4 |
| [1] | | | | | | 500 | 9.5 (120) | 10 | |
| | | | | | | 500 | 11.5 (15) | 50 | |
| | | | | | | 125 | 9.5 (15) | 20 | |
| 17 | $n$-$C_5H_{11}$ | $n$-$C_5H_{11}$ | $CH_3$ | H | 700 | 350 | 9.5 (15) | 95 | 1.4 |
| [1] | | | | | | 350 | 11.5 (15) | 60 | |
| | | | | | | 87.5 | 9.5 (15) | 5 | |
| 18 | $i$-$C_5H_{11}$ | $i$-$C_5H_{11}$ | $CH_3$ | H | 800 | 400 | 9.5 (15) | 94 | 1.45 |
| [1] | | | | | | 400 | 9.5 (120) | 22 | |
| | | | | | | 400 | 11.5 (15) | 70 | |
| | | | | | | 400 | 13.5 (15) | 5 | |
| | | | | | | 100 | 9.5 (15) | 0 | |
| 19 | $CH_3$ | $C_6H_5$ | $CH_3$ | $CH_3CO$ | 900 | 450 | 9 (15) | 93 | 1.35 |
| [8] | | | | | | 450 | 9 (120) | 80 | |
| | | | | | | 450 | 11 (15) | 10 | |
| | | | | | | 112.5 | 9 (5) | 50 | |
| 20 | $i$-$C_5H_{11}$ | $i$-$C_5H_{11}$ | $CH_3$ | $CH_3CO$ | 1000 | 500 | 8.5 (15) | 100 | 1.35 |
| [8] | | | | | | 500 | 8.5 (120) | 90 | |
| | | | | | | 500 | 10.5 (15) | 10 | |
| | | | | | | 125 | 8.5 (15) | 80 | |
| | | | | | | 62.5 | 9 (15) | 20 | |
| 21 | $i$-$C_5H_{11}$ | $i$-$C_5H_{11}$ | H | (a) | 800 | 800 | 7.75 (15) | 80 | 1.3 |
| [9] | | | | | | 800 | 7.75 (90) | 80 | |

| | | | | | | | | | |
|---|---|---|---|---|---|---|---|---|---|
| **22**<br>[9] | $n$-$C_6H_{13}$ | $n$-$C_6H_{13}$ | H | (a) | 1500 | 750<br>750<br>750<br>187 | 7.5 (15)<br>7.5 (90)<br>9.5 (15)<br>7.5 (15) | 100<br>90<br>0<br>40 | 1.5 |
| **23**<br>[6] | $CH_3$ | $p$-$CH_3C_6H_4$ | H | $(CH_2)_2$<br>$CONH_2$ | > 1500 | 1000<br>1000<br>1000 | 8.25 (15)<br>8.25 (90)<br>10.30 (15) | 70<br>60<br>20 | 1.4 |
| **24**<br>[7] | $i$-$C_5H_{11}$ | $i$-$C_5H_{11}$ | H | (b) | 80 | 40<br>40<br>40<br>40<br>10 | 8 (15)<br>8 (90)<br>10 (15)<br>10 (90)<br>8 (15) | 50<br>80<br>0<br>20<br>10 | 1.33 |
| **25**<br>[9] | $n$-$C_6H_{13}$ | $n$-$C_6H_{13}$ | $CH_3$ | H | 1500 | 1000<br>1000<br>1000 | 7.5 (15)<br>7.5 (90)<br>9.5 (15) | 80<br>90<br>80 | 1.5 |
| **26**<br>[7] | $i$-$C_5H_{11}$ | $i$-$C_5H_{11}$ | $CH_3$ | (b) | 150 | 75<br>75<br>75<br>75<br>18.75 | 8 (15)<br>8 (90)<br>10 (15)<br>10 (90)<br>8 (15) | 30<br>90<br>0<br>0<br>40 | 1.3 |
| **27**<br>[7] | $n$-$C_6H_{13}$ | $n$-$C_6H_{13}$ | $CH_3$ | (b) | 200 | 100<br>100<br>100<br>25 | 8 (15)<br>8 (90)<br>10 (15)<br>8 (15) | 60<br>80<br>30<br>40 | 1.3 |

a: $R_4$ =

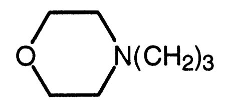

b: $R_4$ =

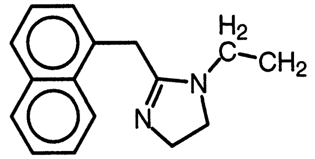

**Method D**

The reaction of bis(diethylamino)dialkylsilanes and –germanes with two equivalents of *N*-substituted cysteamine, methylcysteamine, *N*-(2-thioethyl)-1,3-diaminopropane, *N*-substituted cysteamine or methylcysteamine by naphthylmethylimidazoline and *N*-substituted naphthylmethylimidazoline in anhydrous tetrahydrofuran (a cleavage reaction of M-N bonds by the SH group) gave the corresponding organometallated derivatives, Scheme 4.

$$R_1R_2M(NEt_2)_2 + 2\,HS\underset{\displaystyle R_3}{CH}CH_2\text{-}R_4 \longrightarrow R_1R_2M\Big[S\underset{\displaystyle R_3}{CH}CH_2\text{-}R_4\Big]_2 + 2\,Et_2NH$$

**Scheme 4**

**4. Sila- and germatranes**

$R\text{-}M(OCH_2CH_2)_3N$

Silatranes and germatranes have been synthesized according to two methods already known in the literature [13-18].

**Method E**

This method is the simplest and most general.

The reaction of trimethoxymetallanes, $R\text{-}M(OMe)_3$ (M = Si, Ge), obtained by the action of tetramethoxymetallanes [10] with cysteamine, methylcysteamine and *N*-(2-thioethyl)-1,3-diaminopropane in the presence of triethanolamine leads to corresponding metallatranes (Scheme 5).

**Table II - Radioprotective activity of some selected metalladithioacetals** $R_1R_2M[S\text{-}CH(R_3)\text{-}CH_2R_4]_2$

| M = Si | | | | | | | | | |
|---|---|---|---|---|---|---|---|---|---|
| Cpd [Ref.] | $R_1$ | $R_2$ | $R_3$ | $R_4$ | $LD_{50}$ ($mg.kg^{-1}$) | Injected dose ($mg.kg^{-1}$) | Irradiation Gy (t, min)[a] | Survival rate % | DRF[b] |
| **28** [11] | $CH_3$ | $CH_3$ | H | $NH_2$ | 800 | 400<br>400<br>400<br>100 | 9.5 (15)<br>9.5 (120)<br>11.5 (40)<br>9.5 (15) | 100<br>90<br>40<br>33 | 1.4 |
| **29** [6] | $HCl.H_2N(CH_2)_2S$ | $HCl.H_2N(CH_2)_2S$ | H | (c) | 500 | 250<br>250<br>250<br>62.5 | 7.5 (15)<br>7.5 (90)<br>9.5 (15)<br>7.5 (15) | 60<br>10<br>20<br>30 | 1.3 |
| **30** [12] | *i*-$C_5H_{11}$ | *i*-$C_5H_{11}$ | H | (d) | 100 | 50<br>50<br>50<br>12.5 | 7.75 (15)<br>7.75 (90)<br>9.75 (15)<br>7.75 (15) | 100<br>80<br>10<br>60 | 1.4 |
| **31** [12] | *n*-$C_6H_{13}$ | *n*-$C_6H_{13}$ | H | (d) | 100 | 50<br>50<br>50<br>12.5 | 7.75 (15)<br>7.75 (90)<br>9.75 (15)<br>7.75 (15) | 100<br>100<br>0<br>50 | 1.5 |

a: t = time between administration of compound and irradiation.
b: dose reduction factor = ($LD_{50(30\ days)}$ treated/$LD_{50(30\ days)}$ untreated).

c: $R_4$ =

d: $R_4$ =

| M = Ge | | | | | | | | | |
|---|---|---|---|---|---|---|---|---|---|
| Cpd [Ref.] | $R_1$ | $R_2$ | $R_3$ | $R_4$ | $LD_{50}$ ($mg.kg^{-1}$) | Injected dose ($mg.kg^{-1}$) | Irradiation Gy (t, min)[a] | Survival rate % | DRF[b] |
| **32** [9] | (a) | (a) | H | $NH_2$ | 500 | 250<br>250<br>250<br>62.5 | 7.5 (15)<br>7.5 (90)<br>9.5 (15)<br>7.5 (15) | 80<br>70<br>50<br>0 | 1.4 |
| **33** [11] | *n*-$C_4H_9$ | *n*-$C_4H_9$ | H | $NH_2$ | 600 | 300<br>300 | 9.5 (15)<br>11.5 (15) | 100<br>10 | 1.3 |
| **34** [11] | *n*-$C_4H_9$ | *n*-$C_4H_9$ | $CH_3$ | $NH_2$ | 400 | 200<br>200<br>200<br>50 | 9.5 (15)<br>9.5 (120)<br>11.5 (15)<br>9.5 (15) | 100<br>90<br>20<br>70 | 1.3 |
| **35** [11] | *i*-$C_5H_{11}$ | *i*-$C_5H_{11}$ | H | $NH_2$ | 800 | 400<br>400<br>400<br>100 | 9.5 (15)<br>9.5 (120)<br>11.5 (15)<br>9.5 (15) | 80<br>50<br>60<br>30 | 1.4 |
| **36** [11] | *i*-$C_5H_{11}$ | *i*-$C_5H_{11}$ | $CH_3$ | $NH_2$ | 600 | 300<br>300 | 10 (15)<br>12 (15) | 100<br>95 | >1.6 |
| **37** [11] | *n*-$C_5H_{11}$ | *n*-$C_5H_{11}$ | $CH_3$ | $NH_2$ | 800 | 400<br>400<br>400<br>100 | 9.5 (15)<br>9.5 (120)<br>11.5 (15)<br>9.5 (15) | 100<br>50<br>40<br>40 | 1.4 |

| | | | | | | | | | |
|---|---|---|---|---|---|---|---|---|---|
| **38**<br>[11] | $CH_3$ | $C_6H_5$ | $CH_3$ | $NH_2$ | 600 | 300<br>300<br>300<br>75 | 9.5 (15)<br>9.5 (120)<br>11.5 (15)<br>9.5 (15) | 90<br>20<br>90<br>45 | >1.5 |
| **39**<br>[11] | $CH_3$ | *p*-$CH_3$-$C_6H_4$ | $CH_3$ | $NH_2$ | 800 | 400<br>400<br>400<br>100 | 9.5 (15)<br>9.5 (120)<br>11.5 (15)<br>9.5 (15) | 85<br>70<br>60<br>33 | 1.5 |
| **40**<br>[8] | *i*-$C_5H_{11}$ | *i*-$C_5H_{11}$ | $CH_3$ | $NH(CH_2)_3NH_2$ | 300 | 150<br>150<br>150<br>37.5 | 9 (15)<br>9 (120)<br>11 (15)<br>9 (15) | 87<br>40<br>40<br>90 | 1.4 |
| **41**<br>[9] | *i*-$C_5H_{11}$ | *i*-$C_5H_{11}$ | H | (b) | 900 | 450<br>450<br>450<br>112.5 | 7.75 (15)<br>7.75 (90)<br>9.75 (15)<br>7.75 (15) | 50<br>80<br>0<br>50 | 1.3 |
| ***42***<br>[9] | *n*-$C_6H_{13}$ | *n*-$C_6H_{13}$ | H | (b) | 800 | 400<br>400<br>400<br>100 | 7.5 (15)<br>7.5 (90)<br>9.5 (15)<br>7.5 (15) | 100<br>80<br>0<br>50 | 1.5 |
| **43**<br>[6] | *i*-$C_5H_{11}$ | *i*-$C_5H_{11}$ | H | $NH(CH_2)_2CONH_2$ | > 1500 | 1000<br>1000<br>1000 | 8.25 (15)<br>8.25 (90)<br>10.25 (15) | 100<br>90<br>30 | 1.4 |
| **44**<br>[6] | *n*-$C_6H_{13}$ | *n*-$C_6H_{13}$ | H | $NH(CH_2)_2CONH_2$ | > 1500 | 1000<br>1000 | 8.25 (15)<br>8.25 (90) | 90<br>20 | 1.3 |
| **45**<br>[12] | *i*-$C_5H_{11}$ | *i*-$C_5H_{11}$ | H | (c) | 80 | 50<br>50<br>50<br>50<br>50<br>50<br>12.5 | 7.75 (15)<br>7.75 (90)<br>7.75 (180)<br>9.75 (15)<br>9.75 (90)<br>11.75 (15)<br>7.75 (15) | 100<br>100<br>90<br>70<br>0<br>30<br>80 | 1.7 |
| **46**<br>[12] | *n*-$C_6H_{13}$ | *n*-$C_6H_{13}$ | H | (c) | 150 | 75<br>75<br>75<br>18.75 | 7.75 (15)<br>7.75 (90)<br>9.75 (15)<br>7.75 (15) | 90<br>100<br>10<br>70 | 1.4 |
| **47**<br>[9] | *n*-$C_6H_{13}$ | *n*-$C_6H_{13}$ | $CH_3$ | $NH_2$ | 800 | 800<br>800<br>800 | 7.5 (15)<br>7.5 (90)<br>9.5 (15) | 100<br>70<br>90 | ≥1.6 |
| **48**<br>[7] | *n*-$C_6H_{13}$ | *n*-$C_6H_{13}$ | $CH_3$ | (d) | 160 | 80<br>80<br>80<br>80<br>20 | 8 (15)<br>8 (90)<br>10 (15)<br>10 (90)<br>8 (15) | 60<br>100<br>10<br>0<br>50 | 1.3 |

a: $R_1 = R_2 =$ 

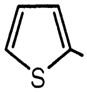

b: $R_4 =$ 

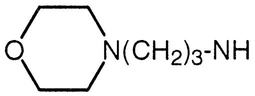

c: $R_4 =$ 

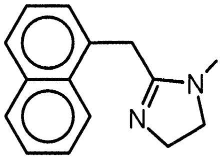

d: $R_4 =$ 

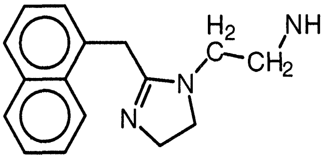

$$M(OMe)_4 + R\text{-}H \xrightarrow[-\,MeOH]{} RM(OMe)_3 \xrightarrow[-\,3\,MeOH]{N(CH_2CH_2OH)_3} R\text{-}M(OCH_2CH_2)_3N$$

**Scheme 5**

**Method F**

The second method of synthesis of silatranes and germatranes includes of methoxymetallatranes. Thus, methoxymetallatranes obtained by the reaction of tetramethoxymetallanes with triethanolamine gave a condensation reaction with cysteamine, methylcysteamine and *N*-(2-thioethyl)-1,3-diaminopropane affording the metallatranes (Scheme 6).

$$M(OMe)_4 + N(CH_2CH_2OH)_3 \xrightarrow[-\,3\,MeOH]{} MeOM(OCH_2CH_2)_3N \xrightarrow[-\,MeOH]{R\text{-}H} R\text{-}M(OCH_2CH_2)_3N$$

M = Si, Ge; R = $-SCH(R')CH_2NH_2.HCl$, $-S(CH_2)_2NH(CH_2)_3NH_2.HCl$; R' = H, $CH_3$

**Scheme 6**

**Table III - Radioprotective activity of some selected metallatranes**
$YHN\text{-}CH_2\text{-}CH(R)\text{-}SM(OCH_2CH_2)_3N$

| Cpd [Ref.] | M | R | Y | $LD_{50}$ ($mg.kg^{-1}$) | Injected dose ($mg.kg^{-1}$) | Irradiation Gy (t, min)[a] | Survival rate % | DRF[b] |
|---|---|---|---|---|---|---|---|---|
| 49 [10] | Si | H | H.HCl | 600 | 300<br>300<br>75 | 9.75 (15)<br>11.75 (15)<br>9.75 (15) | 80<br>10<br>40 | 1.3 |
| 50 [10] | Ge | H | H.HCl | 700 | 350<br>350<br>350<br>87.5 | 9.75 (15)<br>9.75 (120)<br>11.75 (15)<br>9.75 (15) | 80<br>0<br>40<br>0 | 1.4 |
| 51 [10] | Ge | H | $H_2N(CH_2)_3$ | 300 | 150<br>150<br>150<br>37.5 | 9.5 (15)<br>9.5 (120)<br>11.5 (15)<br>9.5 (15) | 60<br>0<br>40<br>10 | 1.4 |
| 52 [10] | Ge | H | 2 $HCl.H_2N(CH_2)_3$ | 900 | 450<br>450<br>450<br>112.5 | 9.5 (15)<br>9.5 (120)<br>11.5 (15)<br>9.5 (15) | 100<br>20<br>30<br>70 | 1.45 |
| 53 [10] | Ge | $CH_3$ | H.HCl | 1500 | 1000<br>1000<br>1000 | 10 (15)<br>10 (120)<br>12 (15) | 80<br>0<br>70 | 1.5 |

**5. Germylated sulfides**

$\{[HCl.H_2NCH_2\text{-}CH(R)\text{-}S]_2GeS\}_3$

**Table IV - Radioprotective activity of germylated sulfides**

| Cpd [Ref.] | R | $LD_{50}$ ($mg.kg^{-1}$) | Injected dose ($mg.kg^{-1}$) | Irradiation Gy (t, min)[a] | Survival rate % | DRF[b] |
|---|---|---|---|---|---|---|
| 54 [9] | $CH_3$ | 1000 | 500<br>500<br>500<br>125 | 7.5 (15)<br>7.5 (90)<br>9.5 (15)<br>7.5 (15) | 100<br>50<br>80<br>10 | 1.6 |
| 55 [10] | H | 800 | 800<br>800 | 9.5 (15)<br>11.5 (15) | 100<br>90 | ≥ 1.5 |

The syntheses of these compounds were realized by the action of NaSH on {[HCl.$H_2NCH_2CH(R)]_2SGeCl_2$ (R = H, $CH_3$) [10] in anhydrous pyridine, Scheme 7.

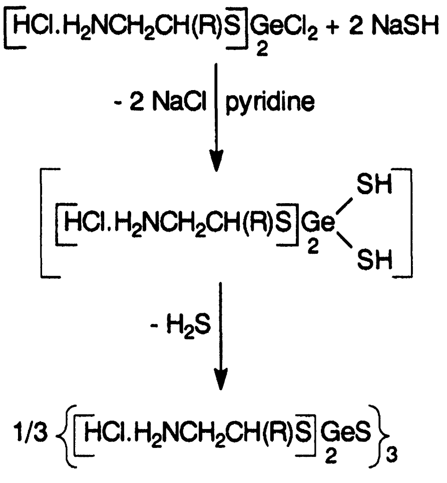

**Scheme 7**

**6. Diisoamyldithiagermocane**

($i$-$C_5H_{12})_2Ge(SCH_2CH_2)_2S$

This compound was obtained by the reaction of 2,2'-thiodiethanethiol with diisoamylgermyldichloride [3], in equimolar amount, in anhydrous tetrahydrofuran and in the presence of freshly distilled triethylamine, Scheme 8.

$$(i\text{-}C_5H_{11})_2GeCl_2 + (HSCH_2CH_2)_2S \xrightarrow{2\ Et_3N} (i\text{-}C_5H_{11})_2Ge(SCH_2CH_2)_2S + 2\ Et_3N.HCl$$

**Scheme 8**

**Table V - Radioprotective activity of diisoamyldithiagermocane**

| Cpd [Ref.] | $LD_{50}$ ($mg.kg^{-1}$) | Injected dose ($mg.kg^{-1}$) | Irradiation Gy (t, min)[a] | Survival rate % | DRF[b] |
|---|---|---|---|---|---|
| **56** [6] | >1500 | 1000<br>1000<br>250 | 7.5 (15)<br>7.5 (90)<br>7.5 (15) | 60<br>50<br>60 | 1.3 |

**7- Di-*n*-hexylpyridinooxathiagermolane**

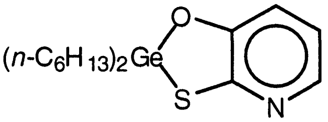

This product was prepared by action of dichlorodi-*n*-hexylgermanium [3] on 2-thio-3-pyridinol in refluxing anhydrous tetrahydrofuran in the presence of freshly distilled triethylamine, Scheme 9.

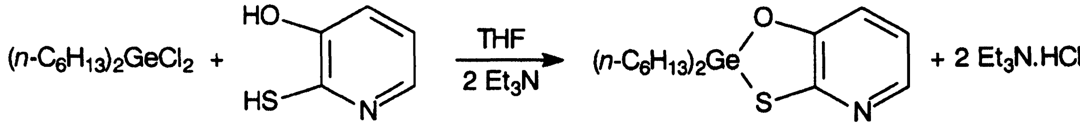

**Scheme 9**

**8. Pharmacology: evaluation of radioprotection**

Male CD1 mice (Charles River, France), 25 g body weight, were used. The compounds were injected intraperitoneally 15, 90, 120 or 180 min before irradiation. The irradiation dose was $LD_{100}/30$ days for untreated mice (7.5, 7.75, 8, 8.25 or 8.3 Gy, according to the irradiation date) or a

2 Gy greater dose. When necessary, other irradiation doses (between 8.5-13.5 Gy were tested in order to evaluate the irradiation $LD_{50}$/30 days of protected mice. The injected dose of the compound was equal to one-half or one-eighth of the $LD_{50}$ value which had been determined previously. The radioprotective effect was evaluated by the Dose Reduction Factor (DRF), which is the ratio between the $LD_{50}$/30 days of treated mice and that of control mice (between 6.5 and 6.75 Gy, according to the date).

**Table VI - Radioprotective activity of di-*n*-hexylpyridinooxathiagermolane**

| Cpd [Ref.] | $LD_{50}$ ($mg.kg^{-1}$) | Injected dose ($mg.kg^{-1}$) | Irradiation Gy (t, min)[a] | Survival rate % | DRF[b] |
|---|---|---|---|---|---|
| 57 [6] | 800 | 400 | 7.5 (15) | 40 | 1.3 |
| | | 400 | 7.5 (90) | 50 | |
| | | 400 | 9.5 (15) | 0 | |
| | | 100 | 7.5 (15) | 20 | |

Irradiation was applied using a cobalt-60 source at the dose rate of 0.3-0.4 $Gy.min^{-1}$ according to the date. During irradiation, animals were placed in a Plexiglass box with 30 cells in a homogeneous field, 28.5 x 28.5 cm in area. Dosimetry was checked with an ionisation chamber dosimeter. The different $LD_{50}$ values were determined by probit analysis.

### 9. Conclusion

The objective of this work was to incorporate potentially radioprotective organic groups in organometallic structures such as metallathiazolidines, metalladithioacetals, metallatranes and germathianes so as to decrease their toxicity and increase their radioprotective activity.

Tables I through VI summarize the radiation protection obtained in mice after intraperitoneal administration in miglyol solution of the organosilylated and organogermylated derivatives described. Generally, these organometallic compounds have a lower toxicity and a radioprotective activity equal to or greater than that of the starting organic derivatives (namely cysteamine [19], methylcysteamine [10] and *N*-substituted cysteamine or methylcysteamine [6, 9] and *N*-substituted naphthylmethylimidazoline [7, 12]). We have to underline that in some cases this great radioprotective activity was obtained with organosilylated or organogermylated derivatives injected in lower doses, expressed in mmol fraction, than those used for starting organic compounds.

The analysis of the toxicity and powerful radioprotective effect of all compounds presented in Table I shows that the metallathiazolidines have generally a good radioprotective activity and lower toxicity compared with starting organic derivatives.

Several compounds in this series (**1**, **2**, **6**, **9** and **10**) exhibit a dose reduction factor (DRF) of 1.5–1.75.

The $LD_{50}$ dose of each compound indicated that they were weakly toxic (500-1500 $mg.kg^{-1}$).

-Derivative **1** (DRF = 1.6; $LD_{50}$ = 800 $mg.kg^{-1}$) at one-half of $LD_{50}$ (the maximum tolerated dose, MTD) this product protects 93 % and 90 % of mice at 10 Gy 15 or 120 minutes after injection and 70 % survival at 12 Gy 15 minutes before irradiation. At one-eighth of the $LD_{50}$, there was a 16 % survival at a dose of 10 Gy.

-Derivative **2** (DRF = 1.5; $LD_{50}$ = 750 $mg.kg^{-1}$) at one-half of $LD_{50}$ 95 % and 89 % protection were obtained at doses of 10 and 12 Gy. At one-eighth of the $LD_{50}$, there was still 75 % survival at a dose of 10 Gy.

-Derivative **6** (DRF = 1.6; $LD_{50}$ = 600 $mg.kg^{-1}$) at one-half of $LD_{50}$ 100 % and 90 % survival were obtained at doses of 7.5 and 9.5 Gy and 50 % survival was observed at a dose of 7.5 Gy. At one-eighth of the $LD_{50}$, there was a 30 % survival at a dose of 7.5 Gy.

-Derivative **9** (DRF = 1.6; $LD_{50}$ = 600 $mg.kg^{-1}$) at one-half of $LD_{50}$ 100 %, 70 % and 40 % survival were obtained at doses of 9.5, 11.5 and 13.5 Gy. At one-eighth of the $LD_{50}$, there was a 50 % survival at a dose of 9.5 Gy.

-Derivative **10** (DRF = 1.75; $LD_{50}$ = 500 $mg.kg^{-1}$) at one-half of $LD_{50}$ 80 %, 70 % and 70 % survival were obtained at doses of 9.5, 11.5 and 13.5 Gy.

The analysis of the results reported in Table II shows that the siladithioacetals and germadithioacetals **31**, **36**, **38**, **39**, **42**, **45** and **47** have an important radioprotective activity (DRF between 1.5-1.7).

-Derivative **31** (DRF = 1.5; $LD_{50}$ = 100 $mg.kg^{-1}$) at one-half of $LD_{50}$ 100 % protection was observed at a dose of 7.75 Gy. At one-eighth of the $LD_{50}$, there was still 50 % survival at a dose of 7.75 Gy.

-Derivative **36** (DRF>1.6; $LD_{50}$ = 600 $mg.kg^{-1}$) at one-half of $LD_{50}$ 100 % and 95 % survival were obtained at doses of 10 and 12 Gy.

-Derivative **38** (DRF>1.5; $LD_{50}$ = 600 $mg.kg^{-1}$) at one-half of $LD_{50}$ 90 % survival was observed at doses of 9.5 or 11.5 Gy. At one-eighth of the $LD_{50}$, there was 45 % survival at a dose of 9.5 Gy.

-Derivative **39** (DRF = 1.5; $LD_{50}$ = 800 $mg.kg^{-1}$) at one-half of $LD_{50}$ 85 %, 70 % and 60 % survival were obtained at doses of 9.5 and 11.5 Gy. At one-eighth of the $LD_{50}$, there was 33 % survival at a dose of 9.5 Gy.

-Derivative **42** (DRF = 1.5; $LD_{50}$ = 800 $mg.kg^{-1}$) at one-half of $LD_{50}$ 100 % and 80 % survival were observed at a dose of 7.5 Gy. At one-eighth of the $LD_{50}$, there was still 50 % survival at a dose of 7.5 Gy.

-Derivative **45** (DRF = 1.7; $LD_{50}$ = 80 $mg.kg^{-1}$) at $LD_{50}$/1.6 100 % and 90 % survival were observed at a dose of 7.75 Gy. 70 % and 30 % survival were also obtained at doses of 9.75 and 11.75 Gy. At $LD_{50}$/6.4, there was still 80 % survival at dose of 7.75 Gy.

-Derivative **47** (DRF>1.6; $LD_{50}$ = 800 $mg.kg^{-1}$) the administration of this product at of $LD_{50}$ protects 100 % and 70 % of mice at a dose of 7.5 Gy and 90 % survival was obtained at a dose of 9.5 Gy.

At last, it is important to note the significant radioprotective activity of organogermylated sulfides **54** and **55** (DRF = 1.5-1.6).

-Derivative **54** (DRF = 1.6; $LD_{50}$ = 1000 $mg.kg^{-1}$) at the MTD this product protects 100 % and 80 % of mice at doses of 7.5 and 9.5 Gy, it also protects 50 % of mice at a dose of 7.5 Gy.

-Derivative **55** (DRF ( 1.5; $LD_{50}$ = 800 $mg.kg^{-1}$) at of $LD_{50}$ 100 % and 90 % protection were observed at doses of 9.5 and 11.5 Gy.

In short, several organosilylated and organogermylated compounds present a good radioprotective activity compared with the starting organic derivatives due to the presence in these molecules of organometallic groups which increase the hydrosolubility and by the presence of organic ligands which increase the liposolubility and biological activity of these molecules, thereby favouring their passage through the cellular membranes.

The results presented in this paper confirm the positive contribution of germanium and silicon in this field in agreement with previous works [1, 6-12, 20-22] and the interesting biological activity of organosilicon and organogermanium compounds in different fields [23-44].

### Acknowledgements

The authors wish to thank the Délégation Générale pour l'Armement (D.G.A.), Département de Chimie-Pharmacologie, Ministère de la Défense Nationale, France, for their financial support and interest in this research.

### References

1 J. Satgé, A. Cazes, M. Bouchaut, M. Fatôme, H. Sentenac-Roumanou and C. Lion, *Eur. J. Med. Chem.*, **17** (1982) 433.
2 G. Dousse, J. Satgé and M. Rivière-Baudet, *Synth. React. Inorg. Met.-Org. Chem.*, **3** (1973) 11.
3 M. Lesbre, P. Mazerolles and J. Satgé in: *The Organic Compounds of Germanium*, John Wiley and Sons, New York, (1973).
4 J. Satgé, M. Lesbre and M. Baudet, *C. R. Acad. Sci. Paris. Ser. C.* **259** (1964) 4733.
5 J. Satgé and M. Baudet, *C. R. Acad. Sci. Paris. Ser. C.* **263** (1966) 435.
6 G. Rima, J. Satgé, H. Sentenac-Roumanou, M. Fatôme, J. D. Laval, C. Lion, C. Thiriot, R. Dagiral and C. Martin, *Main Group Met. Chem.*, **20** (1997) 255.
7 G. Rima, J. Satgé, R. Dagiral, C. Lion, M. Fatôme, V. Roman and J.-D. Laval, *Appl. Organomet. Chem.*, (1999) (under press).
8 M. Fatôme, H. Sentenac-Roumanou, C. Lion, J. Satgé and G. Rima, *Eur. J. Med. Chem.*, **23** (1988) 257.
9 G. Rima, J. Satgé, H. Sentenac-Roumanou, M. Fatôme, J. D. Laval, C. Lion and R. Dagiral, *Appl. Organomet. Chem.*, **10** (1996) 113.

10 J. Satgé, G. Rima, M. Fatôme, H. Sentenac-Roumanou and C. Lion, *Eur. J. Med. Chem.*, **24** (1989) 48.
11 M. Fatôme, H. Sentenac-Roumanou, C. Lion, J. Satgé, M. Fourtinon and G. Rima, *Eur. J. Med. Chem.*, **19** (1984) 119.
12 G. Rima, J. Satgé, R. Dagiral, C. Lion, M. Fatôme, V. Roman and J.-D. Laval, *Metal-Based Drugs,* **5** (1998) 139.
13 M. G. Voronkov and V. P. Baryshok, *J. Organomet. Chem.*, **239** (1982) 199.
14 N. Yu. Khromova, T. K. Gar and V. F. Mironov in: *Heteroorganic Compounds and their applications: Review of Germatranes and their Analogs*, Niitekhim, (1985), Moscow.
15 M. G. Voronkov, *Top. Curr. Chem.*, **84** (1979) 77.
16 M. G. Voronkov, *Pure Appl. Chem.*, **19** (1969) 399.
17 R. Tacke and U. Wannagat, *Top. Curr. Chem.*, **84** (1979) 1.
18 M. G. Voronkov, G. I. Zelchan and E. Lukevics, in Silicon and Life, Berlin Akad., Verlag, (1975).
19 L. Breuil, *La Radioprotection chimique*, Direction des Recherches, Etudes et Techniques, Paris Armées (1979).
20 G. Rima, J. Satgé, M. Fatôme, J. D. Laval, H. Sentenac-Roumanou, C. Lion and M. Lazraq, *Eur. J. Med. Chem.*, **26** (1991) 291.
21 G. Rima, J. Satgé, H. Sentenac-Roumanou, M. Fatôme, C. Lion and J. D. Laval, *Eur. J. Med. Chem.*, **28** (1993) 761.
22 G. Rima, J. Satgé, H. Sentenac-Roumanou, M. Fatôme, J. D. Laval, C. Lion, O. Alazard and P. Chabertier, *Appl. Organomet. Chem.*, **8** (1994) 481.
23 J. Satgé, *Propriétés et applications biologiques de dérivés organométalliques du silicium, du germanium et de l'étain,* rapport de mise au point A. E. P. A., (juin 1981).
24 T. K. Gar and V. F. Mironov, in *Review of the Biological Activity of Germanium Compounds*, Niitekhim (eds), Moscow, (1982).
25 H. A. Meinema, A. M. J. Liebregts, H. A. Budding and E. J. Bulten, *Rev. Silicon Germanium Tin Lead Compd.*, **8** (1985) 157.
26 G. Atassi, *Rev. Silicon Germanium Tin Lead Compd.*, **8** (1985) 219.
27 N. Kakimoto, M. Matsui, T. Takada and M. Akiba, *Heterocycles*, **23** (1985) 2681.
28 T. K. Miyamoto, N. Sugita, Y. Matsumoto, Y. Sasaki and H. Bunkyoku, *Chem. Lett.*, (1983) 1695.
29 J. S. Thayer, *Appl. Organometal. Chem.*, **1**(1987)227; Rev. Silicon, *Germanium Tin Lead Compd.*, **8** (1985) 133.
30 R. Tacke, B. Becker, D. Berg, W. Brandes, S. Dutzmann and K. Schaller, *J. Organomet. Chem.*, **438** (1992) 45.
31 E. Lukevics and L. Ignatovich, *Appl. Organomet. Chem.*, **6** (1992) 113.
32 E. Lukevics, L. Ignatovich, N. Shilina and S. Germane, *Appl. Organomet. Chem.*, **6** (1992) 261.
33 E. Lukevics, S. Germane and L. Ignatovich, *Appl. Organomet. Chem.*, **6** (1992) 543.
34 F. Anger, J. P. Anger, L. Guillou and A. Papillon, *Appl. Organomet. Chem.*, **6** (1992) 267.
35 R. Tacke, D. Reichel, P. G. Jones, X. Hou, M. Waelbroeck, J. Gross, E. Mutschler and G. Lambrecht, *J. Organomet. Chem.*, **521** (1996) 305.
36 E. Lukevics, V. Vevenis and V. Dirnens, *Appl. Organomet. Chem.*, **11** (1997) 805.
37 E. Lukevics, L. Ignatovich and S. Germane, *Khim. Geterotsikl. Soed.*, **10** (1995) 1412.
38 E. Lukevics and L. Ignatovich, *Main Group Met. Chem.*, **1** (1994) 133
39 E. Lukevics, T. K. Gar, L. Ignatovich and V. F. Mironov, *Biological activity of the germanium containing compounds*, Riga, "Zinatne" (1990). (in Russian).
40 F. Li, Z. Zhang, and H. Gao, *Metal Based Drugs*, **5** (1996) 241.
41 E. Lukevics, S. Germane, A. Zidermane, A. Dauvarte, I. M. Kravchenko, M. Trusule, V. F. Mironov, T. K. Gar *et al.*, *Khim. Farm. Zh.*, **18** (1984) 114.
42 E. Lukevics, L. Ignatovich, N. Porsiurova and S. Germane, *Appl. Organomet. Chem.*, **2** (1988) 115.
43 E. Lukevics and S. Germane, L. Ignatovich, *Appl. Organomet. Chem.*, **6** (1992) 543.
44 Proxigermanium (SK-818), *Drugs Fut.*, **18** (1993) 905.

**Received: January 4, 1999 - Accepted: January 25, 1999 - Received in revised camera-ready format: February 5, 1999**

